# Looking for Novel Capsid Protein Multimerization Inhibitors of Feline Immunodeficiency Virus

**DOI:** 10.3390/ph11030067

**Published:** 2018-07-10

**Authors:** Natalia Sierra, Christelle Folio, Xavier Robert, Mathieu Long, Christophe Guillon, Guzmán Álvarez

**Affiliations:** 1 Laboratorio de Moléculas Bioactivas, CENUR Litoral Norte, Universidad de la República, Ruta 3 (km 363), Paysandú C.P. 60000, Uruguay; nataliasierraben@gmail.com; 2 Equipe Rétrovirus et Biochimie Structurale, Université de Lyon, CNRS, MMSB, UMR 5086 CNRS/Université de Lyon, IBCP, C.P. 69367 Lyon, France; folio.christelle@gmail.com (C.F.); xavier.robert@ibcp.fr (X.R.); mathieu.long@ibcp.fr (M.L.)

**Keywords:** assembly inhibitors, immunodeficiency virus, microscale thermophoresis

## Abstract

Feline immunodeficiency virus (FIV) is a member of the retroviridae family of viruses. It causes acquired immunodeficiency syndrome (AIDS) in worldwide domestic and non-domestic cats and is a cause of an important veterinary issue. The genome organization of FIV and the clinical characteristics of the disease caused by FIV are similar to human immunodeficiency virus (HIV). Both viruses infect T lymphocytes, monocytes, and macrophages, with a similar replication cycle in infected cells. Thus, the infection of cats with FIV is also a useful tool for the study and development of novel drugs and vaccines against HIV. Anti-retroviral drugs studied extensively with regards to HIV infection have targeted different steps of the virus replication cycle: (1) disruption of the interaction with host cell surface receptors and co-receptors; (2) inhibition of fusion of the virus and cell membranes; (3) blocking of the reverse transcription of viral genomic RNA; (4) interruption of nuclear translocation and integration of viral DNA into host genomes; (5) prevention of viral transcript processing and nuclear export; and (6) inhibition of virion assembly and maturation. Despite the great success of anti-retroviral therapy in slowing HIV progression in humans, a similar therapy has not been thoroughly investigated for FIV infection in cats, mostly because of the little structural information available for FIV proteins. The FIV capsid protein (CA) drives the assembly of the viral particle, which is a critical step in the viral replication cycle. During this step, the CA protein oligomerizes to form a protective coat that surrounds the viral genome. In this work, we perform a large-scale screening of four hundred molecules from our in-house library using an in vitro assembly assay of p24, combined with microscale thermophoresis, to estimate binding affinity. This screening led to the discovery of around four novel hits that inhibited capsid assembly in vitro. These may provide new antiviral drugs against FIV.

## 1. Introduction

Since its first isolation in 1986, feline immunodeficiency virus (FIV) has been observed worldwide, and FIV infection remains a major health problem among cats, especially in countries with large populations of free-roaming cats [1]. FIV is a lentivirus which causes an immunodeciency syndrome in cats and all the members of the *Felidae* family [2]. FIV closely resembles the human immunodeficiency virus (HIV) in its genomic, biochemical, and morphologic characteristics, as well as clinical and hematological manifestations [3]. As a result, FIV infection of domestic cats is dubbed “feline AIDS” and is considered to be an excellent small animal model for testing prophylactic and therapeutic strategies against human AIDS [4]. A number of antiretroviral drugs for HIV-1, including the prototype nucleoside analogue 3′-azido-3′-deoxythymidine (AZT), have been tested using the FIV model [5]. Several reports have described the in vitro inhibition of FIV by nucleoside-analogue reverse transcriptase inhibitors, such as AZT and 9-(2-phosphonomethoxyethyl)adenine. Moreover, some 4-amino-3,6-disulphonato-l,8-naphthalimide derivatives, such as dextran sulphate, pradimicin A, heparin, phosphonoformate, 2′3′-dideoxythymidine, 2′3′-dideoxyadenosine, and 2′3′-dideoxyinosine, have also been shown to have in vitro activity against FIV in various assays [6]. However, to date, no efficient drug or vaccine for FIV infection has been brought to the market [7].

Recent progress in the combination therapy of several antiretroviral drugs, such as the highly active antiretroviral therapy, has achieved long-term control of HIV replication in vivo [8] and clinical outcome is improved when plasma viral burden is reduced [9]. In the treatment of humans with AIDS, specific targeting of HIV is important. Single-agent treatments are no longer recommended for the treatment of HIV-infected individuals because it promotes the rapid emergence of mutated HIV strains which become resistant to the antiviral agent. Highly active antiretroviral therapy of HIV-infected patients involves the administration of combinations of antiviral drugs from different drug classes, resulting in a slower occurrence of resistant strains [10]. To our knowledge, combination treatment with drugs from different drug classes has not been assessed in FIV-infected cats. This is mainly due to the fact that, despite similarities between the HIV-1 and FIV proteases, all but one of the currently available HIV-1 protease inhibitors failed to inhibit the protease of FIV [11] because of structural differences around the active site [12].

Virion assembly in lentiviruses is the result of a series of steps driven by the multimerization of the structural polyprotein Gag at the plasma membrane of the infected cell. Indeed, the intrinsic biological property of Gag to self-assemble into spherical virus-like particles both in cell cultures or in vitro systems is well documented [4,13,14]. Within the virion, the viral capsid is the protein shell that contains the viral ribonucleoprotein complex, which consists of genomic RNA, nucleocapsid protein, reverse transcriptase, and integrase. Being a lentivirus, lentiviral capsids are conical in shape and consist of a polymer of a single viral protein, the capsid protein (p24 or CA). As in the case of HIV, FIV is assembled as an immature particle, in which CA is released from the Gag polyprotein during proteolytic cleavage by the viral protease and maturation of the particles. After release, CA self-assembles to form the final closed conical structure [15]. Despite that, the CA of FIV represents an interesting therapeutic target which has not yet been exploited.

**Figure 1:**
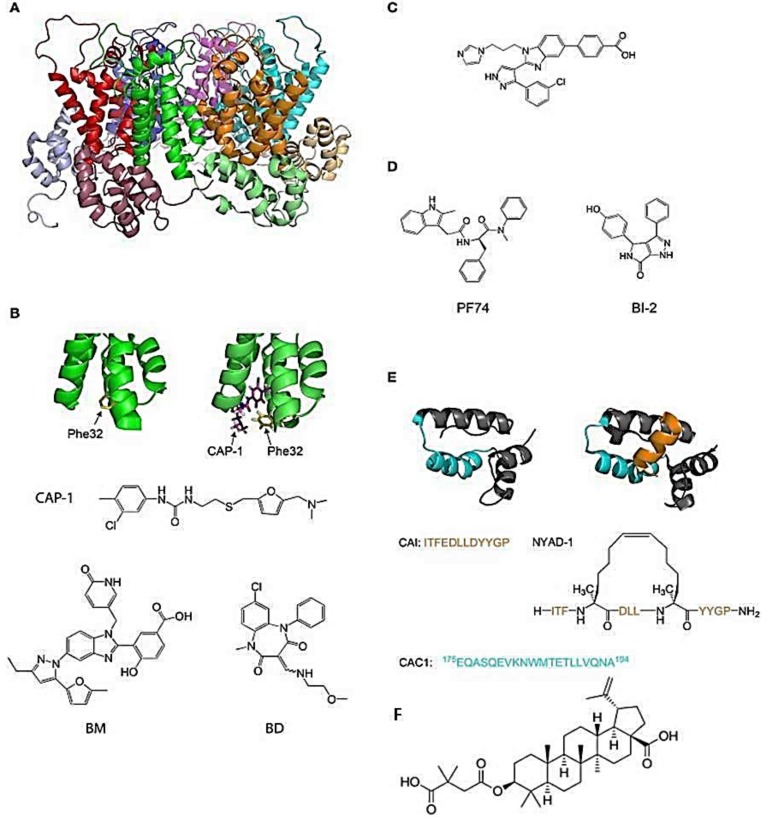
Molecules which inhibit the assembly of HIV-1 viral capsid [16]. (**A**) The structure of the hexamer of the HIV-1 CA protein (PDB ID: 1VUU). The different monomers are green, red, blue, magenta, cyan, and orange. The NTD and the CTD of each monomer are in different shades of the same color; (**B**) The structure of HIV-1 p24 NTD in the absence (**left**) and presence (**right**) of CAP-1. Phe32 is shown in yellow. CAP-1 is shown in magenta (PDB IDs: 1VUU and 2JPR); (**C**) Formula of the CA-binding compound described by Lemke et al. [26]; (**D**) Formula of the CA-binding compounds PF74 and BI-2; (**E**) Structure of the CA protein of HIV-1 CTD in the absence (**left**) and presence (**right**) of the capsid pool inhibitor (CAI, orange). The helix from which the CAC1 peptide was derived is cyan. The sequence of CAI and the structure of the optimized peptide NYAD-1 are given below (PDB IDs: 1AUM and 2BUO); (**F**) The structure of the Bevirimat [9].

The viral capsid plays a crucial role in the first steps of the retroviral infection, including reverse transcription, entry to the nucleus, and integration. In HIV-1, most amino acid substitutions in CA that disrupt the structure and/or stability of the capsid are detrimental to the infection [16,17]. Several host factors interact with CA, and amino-acid substitutions or chemical inhibitors that disrupt these interactions can inhibit HIV infection [18,19]. For example, inhibitors of HIV-1 PF74 and BI-2 (Figure 1D) bind to a pocket between the N-terminal (NTD) and C-terminal (CTD) domains of CA and prevent interactions of the viral capsid with cellular proteins CPSF6 and Nup153 [20,21,22,23]. Additionally, compounds or peptides such as CAP-1 or NYAD-1 (Figure 1B,D), which prevent the formation of the mature functional viral capsid, inhibit the replication of the virus [23,24,25]. An interesting feature of the CA protein as a pharmacological target is its highly conserved sequence in the circulating strains of infected individuals, resulting in a low propensity to develop resistance [26,27]. On the other hand, the inhibitory effect of compound C (Figure 1C) can be reversed with a simple substitution of an amino acid at the N-terminal compound binding site of CA. Thus, the binding site in CA is important because some areas seem more vulnerable to mutations with the concomitant loss of binding to the compound [17]. In another way, inhibition of the capsid assembly acting on DNA control, as done by the drug Bevirimat, produces non-infective viral particles (Figure 1F) [9].

In this work we performed a large screening of around four hundred molecules from our in-house library to identify leads of compounds blocking the assembly of FIV CA. We used an in vitro assembly assay of CA, combined with microscale thermophoresis, to estimate binding affinity, and we used docking studies to get a hint into the protein compounds interfaces. This led to the identification of several lead compounds which warrant further investigation for the development process of new anti-FIV drugs. 

## 2. Results and Discussion

### 2.1. Our In-House Library

**Figure 2:**
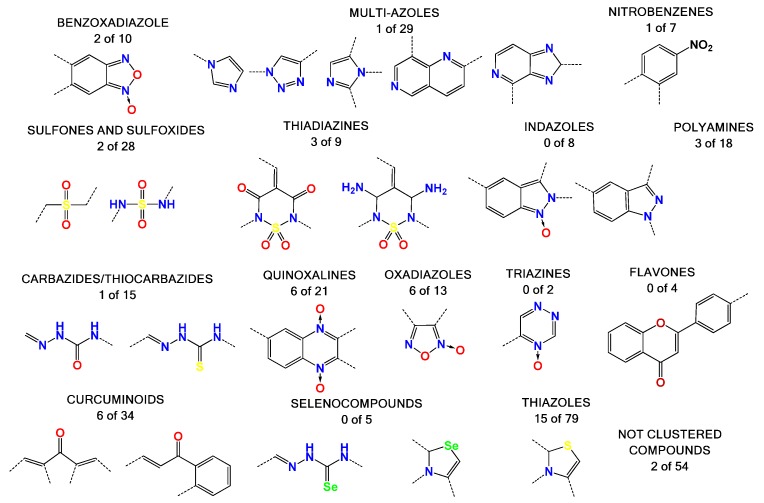
Representation of the compound families used in this work. There are 15 clustered families and 54 compounds with high diversity of structures. The numbers below the families’ names are the molecules with inhibitory effects in our assembly assay versus the total molecules of the family that we tested (see below). For example, we identified two molecules of the benzoxadiazole family which inhibit in vitro assembly out of the 10 molecules tested.

Most of the compounds tested in this study were synthetized previously as part of our ongoing program in drug development for the Chagas disease and other human diseases, such as cancer and aging disease. This resulted in a library containing, to date, more than 2000 compounds [28,29,30]. We selected some compounds which harbored structural similarity with the reported HIV-1 p24 assembly inhibitors, as well as compounds randomly selected to representing all of the chemical families present in our library (Figure 2). A database with all the compounds from our library was generated using the ChemOffice^®^ 12.0 software package (Cambridge, MA, USA). Then, structural similarities with known HIV capsid inhibitors were estimated using the ChemFinder tool from ChemOffice^®^. For the selection process, we used a structural similarity criteria of 80% with the reported inhibitor of CA as a search filter (Supplementary Figure S1). As an example, this resulted in the identification of 18 molecules from our library which harbor 80% similarity with the CAP-1 HIV-1 inhibitor (Figure 1), from which the compound with the best score was selected [9,22,23,24,31,32,33]. The random selection of the other compounds was performed based on several criteria: abundance of the compound, solubility, cost-effective synthetic procedures, and compounds with toxicology data. The structural details of the selected molecules are in Table S1 (Supporting Information), with some examples in Figure 2, and include families like benzofuroxanes, furanes and thiophenes, 4-substituted-1,2,6-thiadiazines and its synthetic precursors, quinoxaline 1,4-dioxides, phenazine 5,9-dioxides, furoxanes, imidazole *N*-oxide, indazoles, thiazoles, 1,2,4-triazine *N*-oxides, flavonoids, nitroalkenes, and others. 

### 2.2. In Vitro Assembly Studies

To find novel chemical structures as a backbone for the design of new antiviral drugs targeting the capsid assembly of the virus, we optimized a CA assembly in vitro assay for screening purposes [34]. 

Indeed, our initial assay used light scattering and could only monitor the assembly of one sample per experiment on 40 min kinetics [35]. This resulted in using almost 20 mg of protein in a 14 h period to test 10 compounds, in duplicate, using light scattering, which was not adequate for screening methods. The use of the spectrometric technique to follow capsid assembly has been described for HIV [24] and is based on the detection of capsid assembly via the measurement of the increase of medium turbidity. The use of this technique to monitor FIV p24 assembly allowed us to use 384-well plates, reducing the volume of protein to 15 µL per sample and allowing a testing of 10 compounds, in duplicate, in 40 min. Moreover, in this format, the protein concentration (5 mg/mL) was enough to start the assembly without needing the high concentration of salt used in the HIV in vitro assembly assay. High salt concentration could cause a problem by interfering with the solvation and solubility of some molecules and the loss of their interaction with the target. The only limitation of this method using low volumes was the risk of introducing air bubbles in the reaction, which interfere negatively in the absorbance measurement. However, this issue was solved by a careful manipulation in the filling process of the plates. 

The most important limitations in screening our compounds in the assembly assay were the dimethyl sulfoxide (DMSO) concentration. In the case of the CA assembly, the maximal concentration of DMSO was 0.25–0.5% *v*/*v*. This makes sense because the assembly process depends on a weak interaction of the protein surface. As DMSO is a surfactant, presence of DMSO might “solubilize” the CA monomers. However, this maximal DMSO concentration is too low for several lipophilic molecules, and many molecules could not be tested for that reason. To get better solubility, we replaced DMSO with glycerol for some compounds which, in some cases, was useful but was not sufficient to solubilize some compounds. 

**Figure 3:**
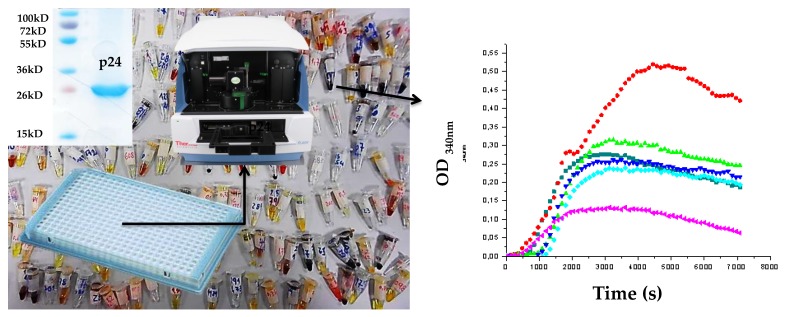
Left panel: general representation of the optimized screening in the assembly assay. Purity of p24 is shown in the inset. Right panel: two discriminable behaviors are visible compared to the control (in dark blue). In red is the enhancement of the process, and in pink is the inhibition of the process. The green and light blue curves are typical for compounds with no effect on the p24 assembly.

With this spectrometric assembly assay, we were able to discriminate two different behaviors for compounds interfering with the assembly of FIV CA: the enhancement of the assembly or its inhibition. The enhancement of assembly is associated with an increase of the diameter of the assembled objects and could result in a disordered or altered capsid with a loss of function. Inhibition is the classical effect which would impair the formation of the capsid (Figure 3). 

The compounds were selected as an active compound when the inhibition of the assembly process was more than 40% at 50 or 25 µM. The oxadiazoles family was the most active, with 50% of its members showing assembly inhibition activity. High nitrogen content heretocycles were not a good source of bioactive molecules. Some compounds with structural similarities with Bevirimat were also active, like some steroid compounds clustered in diverse families. The thiazoles family shows several active compounds, but these are highly diverse and do not show a structural correlation between them. On the other hand, compounds of the thiadiazine family showed active molecules with structures that are closely correlated. We could not find active molecules in four families (i.e., selenium containing compounds, flavones, triazines, and indazoles).

The dose response could not be calculated with this method. To confirm the interaction of our compound with FIV CA and get a dose response, we performed microscale thermophoresis (MST) to confirm the interaction of the active compounds and their affinity for CA. 

### 2.3. Microscale Thermophoresis Studies

**Figure 4:**
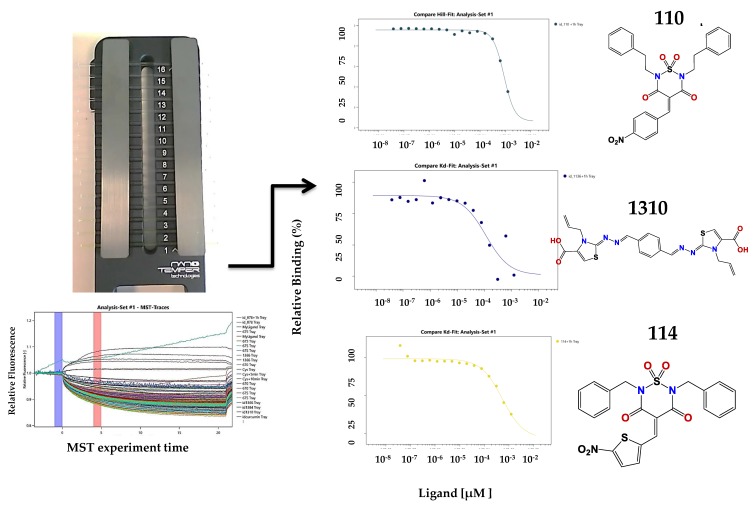
Left panel: schematic representation of the MST assay. It is the cassette with 16 capillary spaces to get 16 points in the doses response assay. On the right are some of the results obtained.

**Figure 5:**
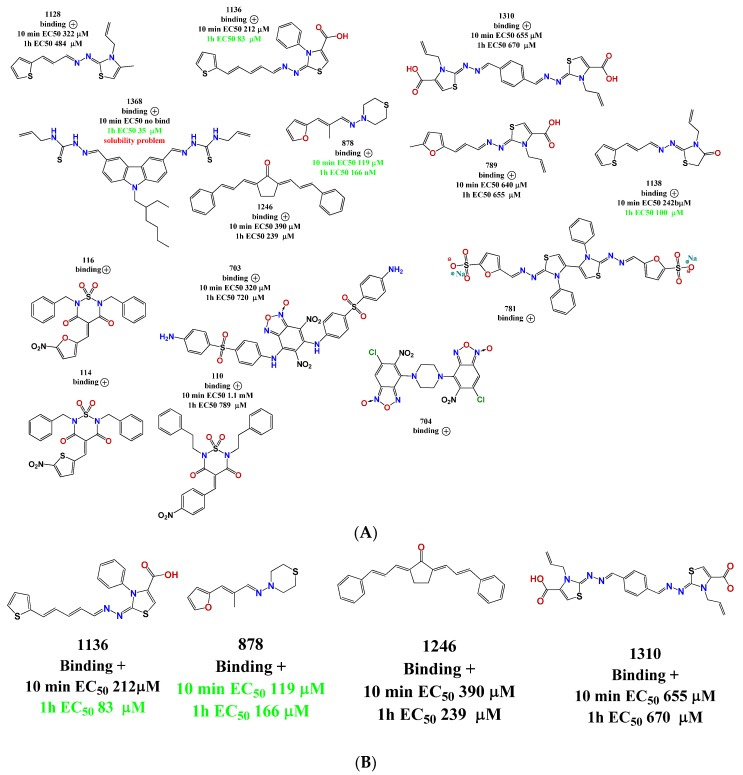
(**A**) Compounds binding to CA identified in the MST screening. With each structure are the binding data and the EC_50_ values; (**B**) Verified hits in the polymerization assay.

For the active compounds selected from the screening, we checked the binding affinity using MST (Figure 4). Microscale thermophoresis can cope with the presence of 5% of DMSO, resolving the solubility problems that we encountered in the assembly assay for some compounds. Another advantage of MST is that it uses low concentrations of protein (in the nanomolar range), which could be better in analyzing the interaction process because the compound/protein ratios were inverted compared to the assembly assay. The major disadvantage of this method is the interference of the compound with the measurement, either by autofluorescence or quenching. However, from the 48 active compounds which we identified by spectrometric methods, we confirmed using MST that 14 compounds bind to p24 (Figure 5).

We explored the binding affinity at two different times (10 and 60 min) to enhance the poetical interactions. There is a correlation between the time and the binding potency for some of the molecules. The most active compound was thiazole **1136** with an EC_50_ of 83 µM, in comparison with the affinity of one of the first reported assembly inhibitor of HIV-1 compounds, CAP-1 (EC_50_ = 60 µM), which was active in the in vitro infection assay with HIV-1 at 100 µM [24].

A structure motif was repeated in some active molecules, such as carboxylic groups and sulfoxides, granting a further analysis of these families of molecules. These 14 compounds were validated again in our assembly assay by light scattering and spectrophotometry. From these, four molecules were validated with a binding affinity to CA and inhibition of the assembly: compounds **878**, **1136**, **1246**, and **1310** (Figure 5B). Also interestingly, these same molecules were used in the screening of five different enzymes from pathogen parasites but did not display inhibitory effects at 100 µM [36,37], suggesting that our compounds are not unspecific interactors.

### 2.4. Docking Analysis

**Figure 6:**
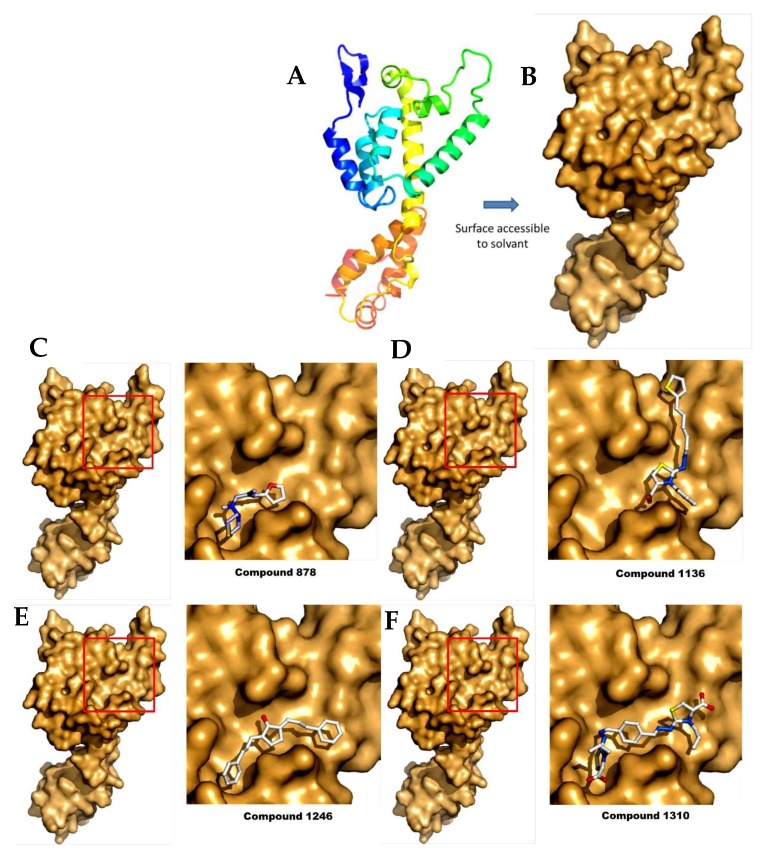
Representation of the CA monomer and the different docking studies from the selected compounds. (**A**) The monomer of CA in cartoons; (**B**) The CA surface accessible to the solvent, computed using PyMol. The best scored positions after docking on CA (**left panel**), with a close-up view (**right panel**) of the binding region (red square) for (**C**) compound **878**; (**D**) compound **1136**; (**E**) compound **1246**; and (**F**) compound **1310**.

To study the different binding modes of these compounds, we performed some computational studies by docking. Surprisingly we found that the compounds bind on the same surface of the protein in the 25-Å-long ridge comprised between helices a4 and a7, although each compound showed a specific binding site. Although this surface is not directly involved in HIV assembly, it is close to the monomer:monomer interface in the published HIV CA hexamer (Figure 6) [38]. Moreover, a recent study identified residues of the FIV capsid, which are important for FIV capsid assembly and are located in helix 4. Although our compounds do not bind directly to these two residues, these compounds could change the conformation of this region upon binding, resulting in the inhibition of p24:p24 interactions during capsid assembly [39].

For each compound, the ten best docking positions were sorted based on their predicted ΔG binding values. Interestingly, predicted K_D_ values deducted from the dockings are also in the µM range (Table 1). In particular, the K_D_ value of compound **1136** was predicted to be between 17 and 36 µM, while showing an experimental value of 83 µM when measured by MST (see above).

### 2.5. Toxicology Data

**Figure 7:**
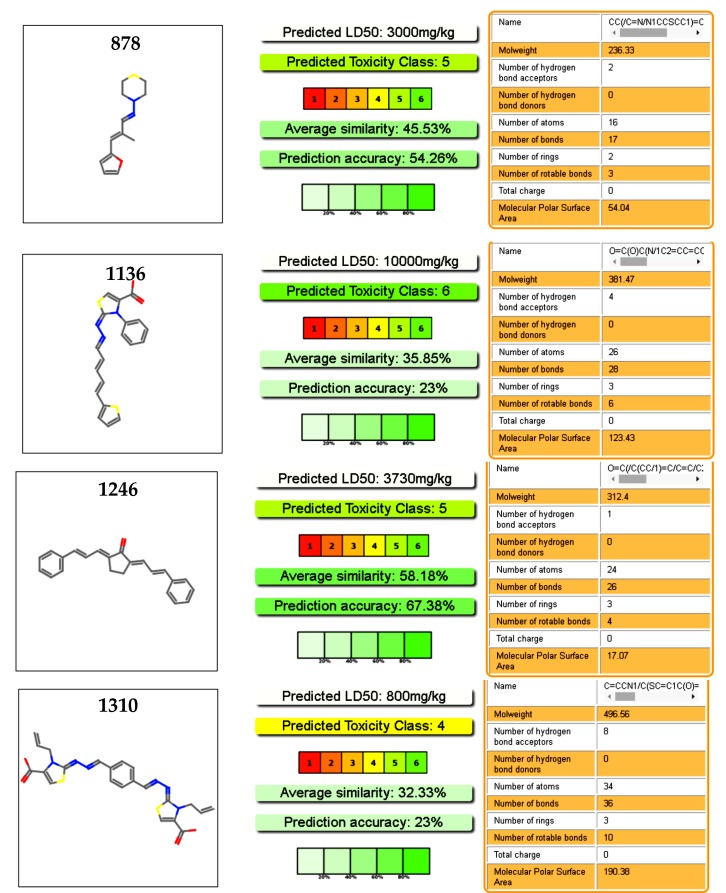
Toxicology profiles of the studied compound calculated from PROTOX (Charite University of Medicine Institute for Physiology Structural Bioinformatics Group Philippstrasse 12, 10115 Berlin, Germany) [40].

We explored the toxicology profiles of the active molecules using an online predictor with a database of a hundred thousand molecules. Our selected molecules were considered safe drugs, as deduced from the prediction of the oral toxicity in mice (Figure 7). Cytotoxicity predictions of hepatic toxicity and genotoxicity were also low for these molecules. In the case of **878** and **1246**, cytotoxicity predictions were corroborated by our previous work on these molecules, with a cytotoxicity in murine macrophages of IC_50_ > 100 µM [37]. Moreover, the four hits were assayed with two different type of mammalian cell at 100 µM, without cytotoxic effect at this dose (Table S2) [29]. Compound **1246** was used in a murine acute infection model of the Chagas disease and was administrated at 100 mg/kg body with for 15 days, without toxic effect during this period [28].

## 3. Experimental Section

### 3.1. Expression and Purification of CA

The FIV CA protein was expressed and purified mostly as described [35,39]. *Escherichia coli* cells (BL2I (DE3) pLysS, Lucigen, Middleton, WI, USA) transformed with pRSET-p24EDCP-T were grown in Lysogenic broth medium (Sigma-Aldrich, Saint-Quentin-Fallavier, France), supplemented with 50 mg/mL of ampicillin, at 37 °C. When cells reached an OD_600_ value between 0.3 and 0.4, the expression of CA was induced by the addition of 1 mM isopropyl-α-d-1-thiogalactopyranoside (IPTG, Euromedex, Souffelweyersheim, France) for 3 h at 37 °C, then cells were harvested by centrifugation and the pellets were stored overnight at −20 °C. The purification of p24 protein was performed by nickel affinity chromatography, as described [40], using batch incubation with Ni^2+^-TED resin (Macherey-Nagel, Hoerdt, France)), followed by loading onto a gravity column. The column was washed three times with LEW buffer (50 mM NaH_2_PO_4_, 300 mM NaCl, pH 8.5), and the elution was performed with LEW buffer containing 50 mM of imidazole. The concentration of CA was quantified by spectrophotometry at 280 nm, using a Nanodrop (Thermo Fisher, Waltham, MA, USA). The purity of the protein at homogeneity was checked using SDS-PAGE analysis before further processing (Figure 3). Buffer exchange, using ultrafiltration devices (10 kD MWCO, Sartorius, Aubagne, France), was performed against a HEPES/NaCl Buffer (50 mM HEPES pH 6.5, 100 mM NaCl).

### 3.2. Assembly Assay by Spectrophotometry [24]

The CA assembly assay was performed in a final volume of 50 μL in 384-well plates, with a final concentration of 5 mg/mL of purified recombinant FIV p2 in 50 mM NaH_2_PO_4_ and 1 M NaCl, pH 7.4. Absorbance is measured at 340 nm every 30 s in a multiplate reader Varioskan^TM^ Flash Multimode Reader (Thermo Scientific^TM^, Waltham, MA, USA) at 38 °C for 30 min. The compounds were tested at a fixed initial dose of 25 or 50 μM (at 0.5% DMSO *v*/*v*) by adding 1 µL of the concentrated ligand were added to the well before starting the measurement. The controls were CA at 5 mg/mL in only 0.5% DMSO *v*/*v* and CA without DMSO. The criteria to select active compounds were the observation of at least 50% of assembly inhibition at these doses, based on the reduction of the OD value at 340 nm at the plateau value compared to the controls.

### 3.3. Microscale Thermophoresis [41]

MTS experiments were performed according to the NanoTemper technologies protocol in a Monolith NT.115 (green/blue) instrument (NanoTemper Technologies, München, Germany). With this technique, the diffusion behavior of a labeled protein is measured when an infrared light excites the movement of the protein in capillaries. This behavior is the combination of two effects: the fast, local environment dependent responses of the fluorophore to the temperature jump and the slower diffusive thermophoresis fluorescence changes. It will be modified when the protein is complexed with increasing amounts of unlabeled partners, leading to titration curves that can be fit for Kd estimation [41]. In practice, CA was labeled with the Monolith His-Tag Labeling Kit RED-tris-NTA (NanoTemper Technologies, München, Germany), as described by the manufacturer. The experiments were performed using 20% and 40% MST power and between 20–80% LED power at 24 °C. The MST traces were recorded using the standard parameters: 5 s MST power off, 30 s MST power on, and 5 s MST power off. The compounds were used at high concentrations (around 5 mM) in the bindings check assay with DMSO at 5% *v*/*v*. If the binding check was positive, then the affinity determination was performed with the same experimental settings, with serial 2× dilutions of the compound of interest.

### 3.4. Docking

The crystal structure of the full-length FIV CA that we previously solved (PDB entry 5NA2) [40] was used as a target protein in the subsequent docking experiments. In this PDB entry, CA NTD and CA CTD domains are in a compact form, due to an artifactual intermolecular disulfide bond. In order to obtain its open form, we used the hexameric crystal structure of the HIV-1 capsid protein CA as template (PDB entry 5HGN) [38]. CTD and NTD domains from FIV CA were superimposed to those of HIV-1, and the linker region (residues 137–141) was reconstructed manually using COOT [42], respecting the backbone and side-chains conformational restraints.

Compounds were modelled using the smiles code from the Chemoffice software. Then, CA in its open form and compounds were prepared using AutoDockTools v1.5.6 [43]. The polar hydrogen atoms were added, the non-polar hydrogens were merged, and the Gasteiger partial atomic charges were computed. Finally, all the possible rotatable bonds were assigned for each compound molecule.

A “blind docking” was then carried out with the program AutoDock Vina v1.1.2 [44]. Compounds were treated as flexible while the target protein was treated as rigid. The search space was defined in order to encompass the entire surface of the target protein, and a large exhaustiveness value of 1000 was used regarding the wide search space (76 × 60 × 44 Å^3^). A visual examination of the resulting docking poses was carried out using PyMOL 1.5 (Schrödinger, Delano Scientific, LLC, New York, NY, USA).

### 3.5. Cytotoxicity Assay in Mammalian Cells

Vero cell lines (ATCC, Rockville, MD, USA) were cultivated in Dulbecco’s modified Eagles medium (DMEM) with 10% FCS in a humid atmosphere of 5% CO_2_ at 37 °C. Vero cells were seeded in 96-well plates at an initial density of 5 × 10^3^ cells per well. J774.1 murine macrophage cells (ATCC, Manassas, VA, USA) were grown in DMEM culture milieu containing 4 mM l-glutamine and supplemented with 10% FCS. The cells were seeded in a 96-well plate (5 × 10^4^ cells in 200 µL culture medium) and incubated at 37 °C in a 5% CO_2_ atmosphere for 48 h, to allow cell adhesion prior to drug testing. Both cell lines were exposed for 48 h (at 37 °C and 5% CO_2_) to the compounds (100 µM) or the solvent (0.4% DMSO) for the control, and additional controls (cells in medium) were used in each test. The cytotoxicity of the compounds was evaluated by MTT reduction assays. To proceed with the MTT assay, 20 μL of 3-(4,5-dimethylthiazol-2-yl)-2,5-diphenyl-2*H*-tetrazolium bromide (MTT) 5 mg/mL solution dissolved in 1× PBS was added to the wells and incubated for 4 h at 37 °C in a 5% CO_2_ controlled atmosphere. Next, the medium was aspirated and 100 μL of DMSO was added to each well and incubated at room temperature in the dark for 15 min with moderate orbital shaking. Optical density (OD) was read in a plate spectrophotometer (Thermo Scientific Varioskan^®^ Flash Multimode, Waltham, MA, USA) at 570 and 690 nm wavelengths. Each experiment was performed in triplicate. For the statistical analyses, the GraphPad Prism 6 was used. Dunnett’s multiple comparison tests and Student’s *T*-test were used when we compared the averages of each condition with the control [45].

## 4. Conclusions

In this work, we performed a large screening of 400 molecules from a large library of compounds, looking for new hits for antiretroviral drug development against the FIV assembly. For this, we optimized methods towards a cost-effective screening method for FIV assembly inhibitors. Then, we combined the complementary methods using the CA protein (i.e., assembly assay, MST, and docking), allowing the identification of four compounds which represent interesting leads for the design of CA assembly disrupters. Interestingly, these compounds also showed no cytotoxic effects in mammalian cells, thus representing interesting candidate molecules which will now be tested in in vitro infection assays with live viruses.

## Figures and Tables

**Table 1:** Data from docking studies with the active compounds identified in the in vitro assay with CA.

Compounds	Predicted Binding ΔG (kcal·mol^−1^) Range Values	Predicted KD (µM) Range Values
**878**	−5.2/−4.9	163/269
**1136**	−6.6/−6.1	17/36
**1246**	−7.4/−7.0	4/8
**1310**	−6.3/−6.0	26/43

## Supplementary Materials

The following are available online at http://www.mdpi.com/1424-8247/11/3/67/s1, Table S1. Complete list of molecules assayed in the CA assembly. Table S2. Cytotoxicity assay in mammalian cells. Figure S1. Output of the search using ChemFinder from ChemOffice software.
