# Supplementary material for: Looking for Novel Capsid Protein Multimerization Inhibitors of Feline Immunodeficiency Virus
Source: Pharmaceuticals (Basel). 2018 Jul 10;11(3):67. doi: 10.3390/ph11030067 (PMC6161179; doi:10.3390/ph11030067)

Table S1. Complete list of molecules assayed in the CA assembly.

Sample N^er	ID	Structure	Assembly	Binding
**BENZOXADIAZOLE**				
1	10	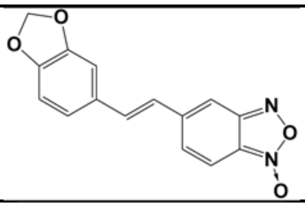	— *	nd**
2	25	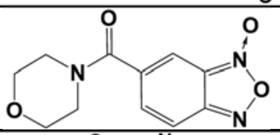	—	nd
3	27	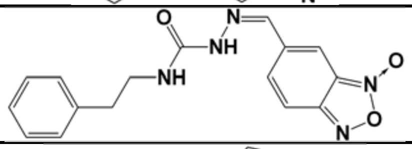	—	nd
4	598	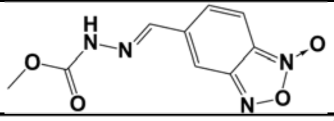	—	nd
5	609	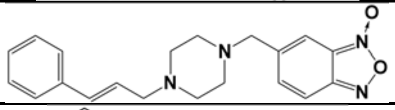	—	nd
6	615	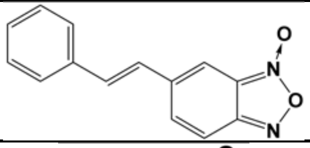	—	nd
7	627	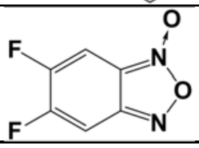	—	nd
8	38		—	nd
9	704	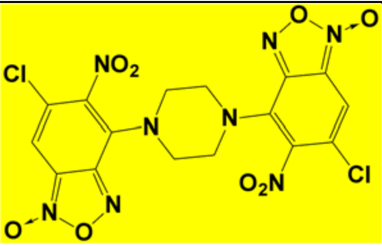	—	+ *
10	1398		nd	+
**SULFONES AND SULFOXIDES**				

11	187	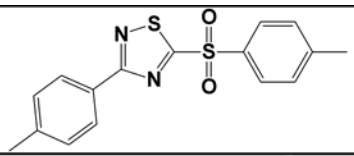	—	—
12	72	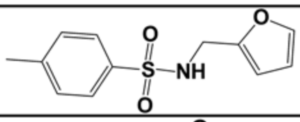	—	nd
13	124	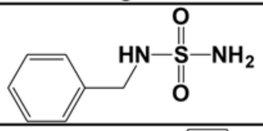	—	nd
14	118	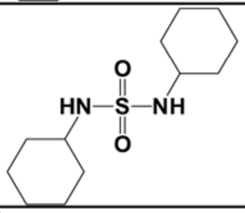	—	nd
15	119	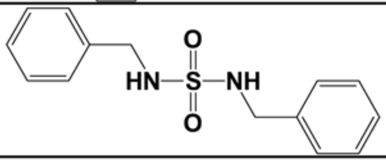	—	nd
16	120	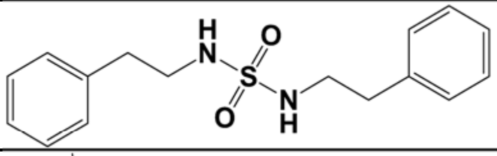	—	nd
17	121	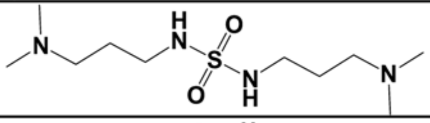	—	—
18	122	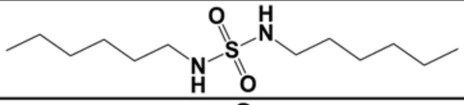	—	nd
19	123	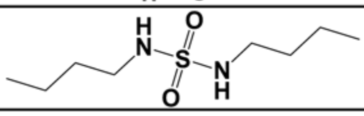	—	nd
20	120		—	nd
21	1369	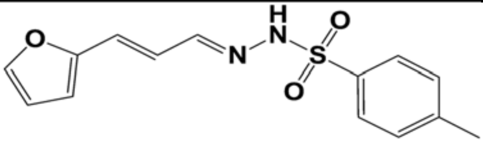	nd	—
22	707	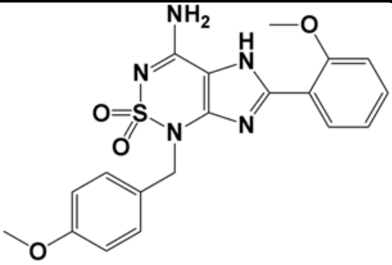	—	nd

23	703	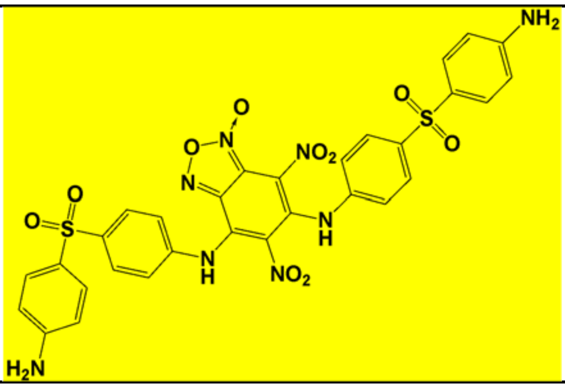	+	+
24	MMV688 180			
25	MMV658 993			
26	MMV023 860			
27	MMV010 576	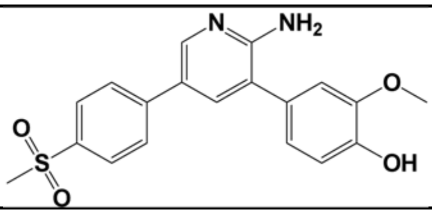	—	nd
28	MMV020 291			
29	MMV687 246			
30	MMV024 114			

31	MMV006833			
32	MMV022029		—	nd
33	1121		—	nd
34	MMV688467			
35	1407		nd	+
36	MMV676050			
37	MMV001059			
38	MMV007133			

39	MMV024 101			
**THIADIAZINES**				
40	126	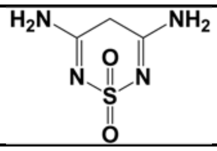	—	nd
41	723		—	nd
42	127	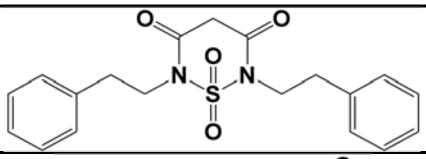	—	nd
43	117	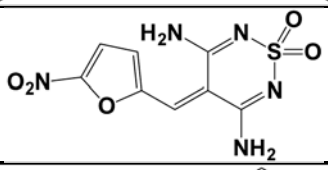	—	nd
44	111	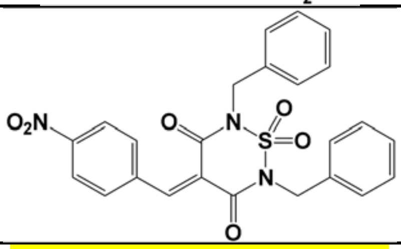	—	nd
45	114		nd	+
46	113	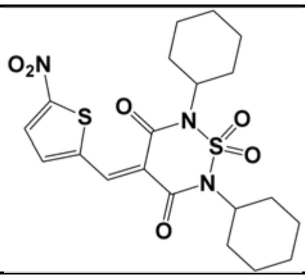	—	nd
47	128		nd	—

48	110		nd	+
49	116		nd	+
**MULTI-AZOLES**				
50	45	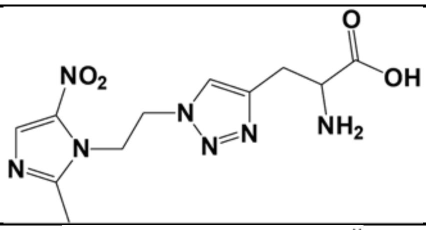	—	nd
51	1384	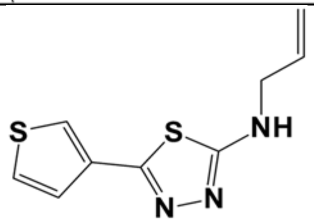	nd	—
52	MMV022 478			
53	637	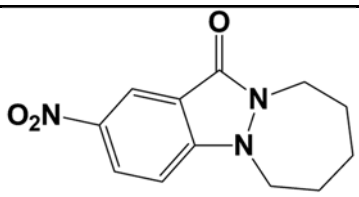	—	
54	334	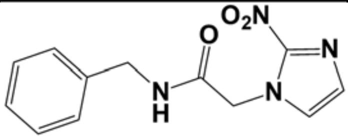	—	nd
55	710	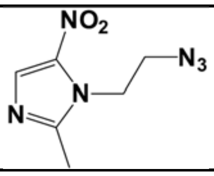	—	nd

56	712	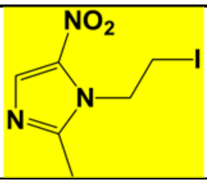	+	nd
57	MMV020670	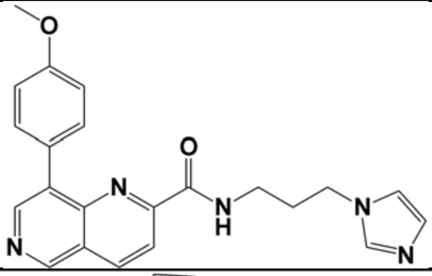	—	nd
58	MMV032967	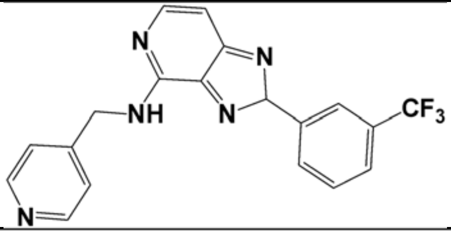	—	nd
59	MMV031011	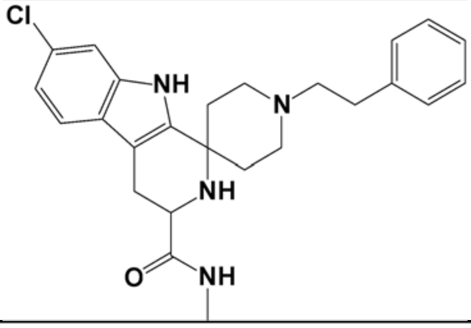	—	nd
60	MMV688178	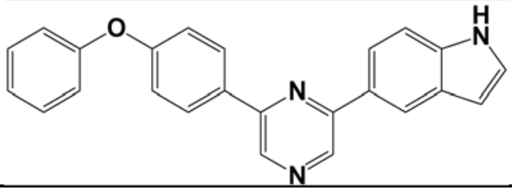	—	nd
61	MMV688362		—	nd
62	MMV687706		—	nd
63	MMV026356		—	nd

64	MMV007 625		—	nd
65	532		—	nd
66	MMV045 105			
67	MMV688 407			
68	MMV023 949			
69	MMV688 474			
70	109	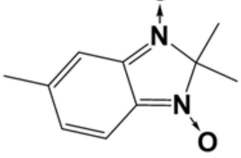	—	nd

71	553	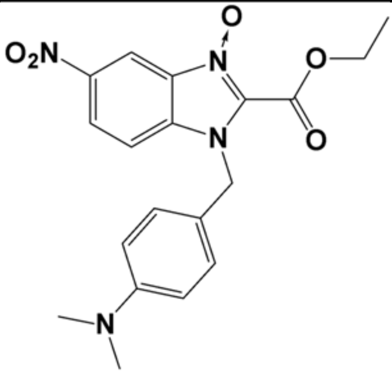	—	nd
72	1278		nd	—
73	1366	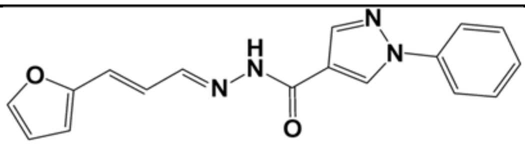	nd	—
74	MMV009135			
75	MMV011691			
76	MMV663250			
77	MMV407539			

78	MMV688 372			
79	MMV676 182			
**OXADIAZOLES**				
80	165	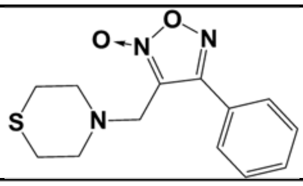	—	nd
81	175	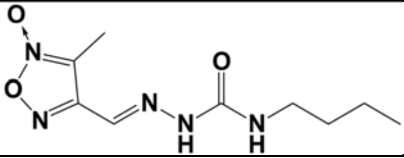	—	nd
82	1253		nd	—
83	173	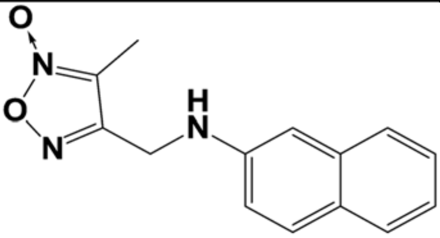	—	
348	1183		nd	+
84	1182		nd	+

85	1184		nd	—
86	1191		nd	—
87	1283		nd	+
88	1213		nd	+
89	1210		nd	+
90	1207		nd	—
91	1203		nd	+
92	1199		nd	—

INDAZOLES

93	198	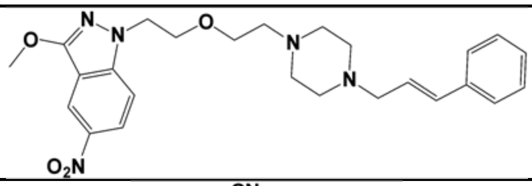	—	nd
94	202	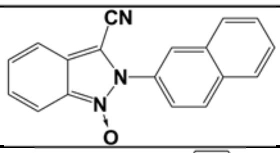	—	nd
95	203	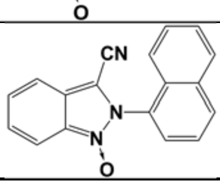	—	nd
96	205		—	nd
97	459	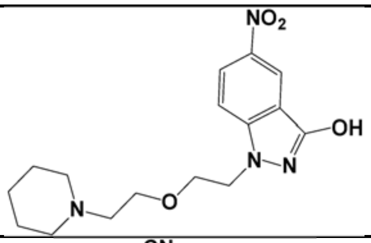	—	nd
98	468	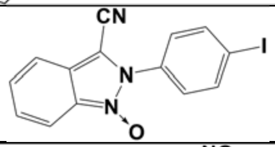	—	nd
99	514	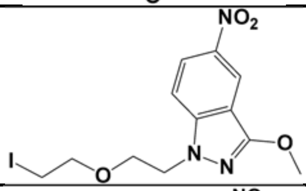	—	nd
100	517	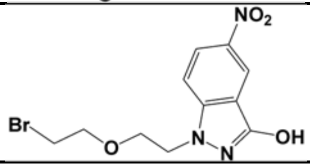	—	nd
101	482		—	nd

QUINOXALINES

102	207	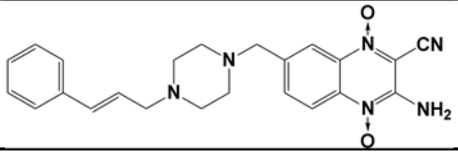	—	nd

103	208	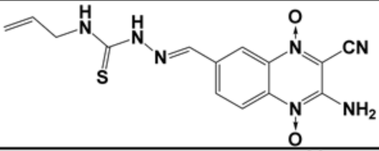	—	nd
104	492	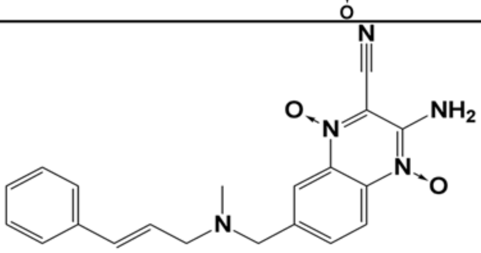	—	
105	327	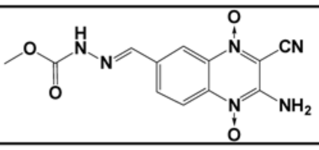	—	nd
106	302		—	nd
107	655	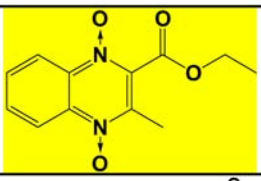	+	—
108	221	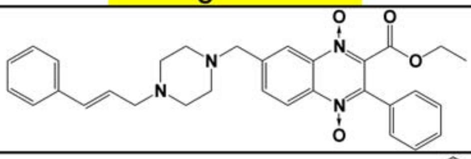	—	nd
109	297	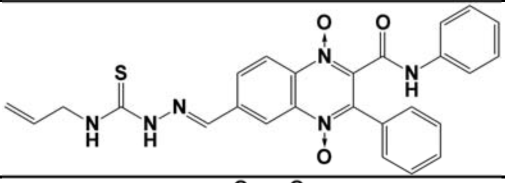	—	nd
110	653	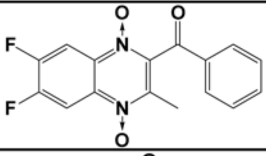	—	nd
110	473	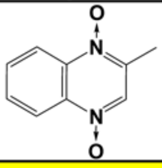	—	nd
112	298	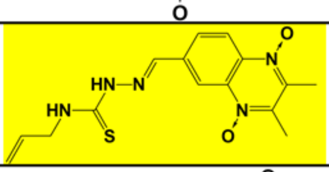	+	nd
113	362	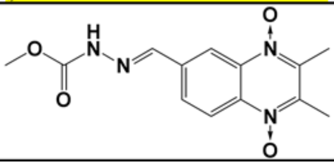	—	nd

114	364	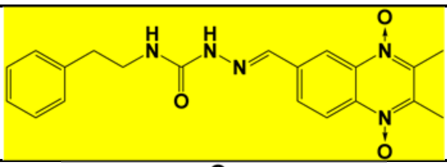	+ / −	nd
115	656	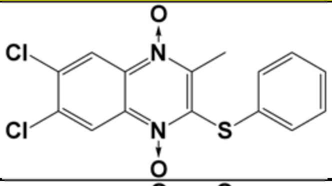	−	nd
116	658	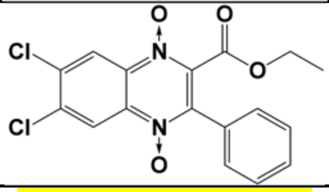	−	nd
117	1400		−	+
118	680	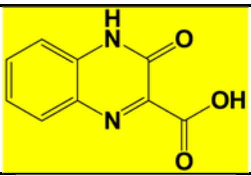	+	−
119	681	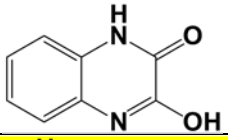	−	nd
120	687	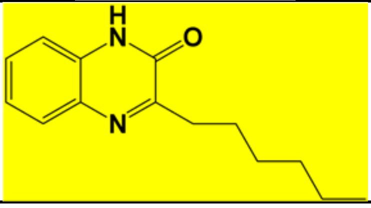	+	−
121	684		−	nd
122	656		−	nd

123	303		—	nd
**NITROBENZENES**				
124	243	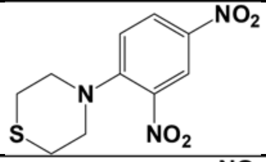	—	nd
125	244	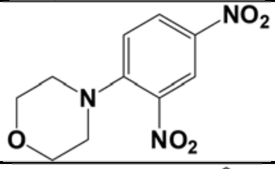	—	nd
126	245	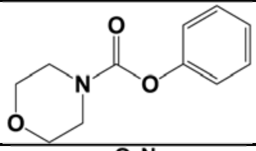	—	nd
127	248	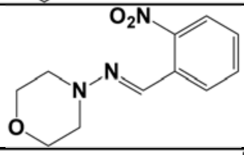	—	nd
128	569	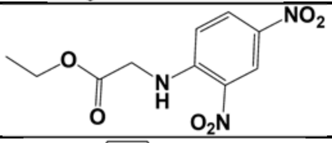	—	nd
129	631	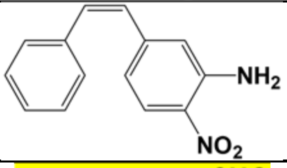	—	nd
130	670	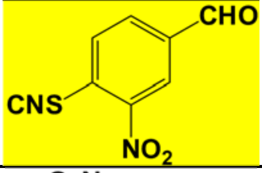	ENHANCER	+
131	705	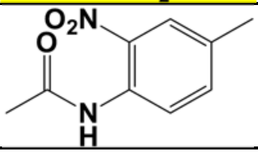	—	nd
**CARBAZIDES**				
132	353	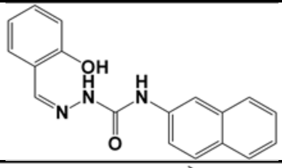	—	nd
133	387	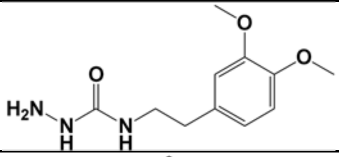	—	nd
134	701	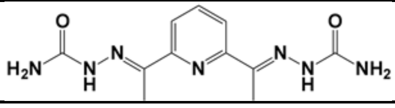	—	nd

135	1367	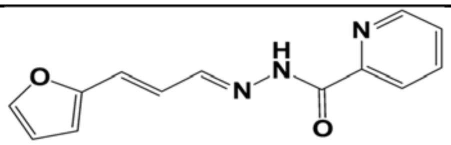	nd	—
**THIOCARBAZIDES**				
136	291	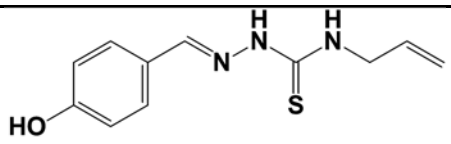	—	nd
137	296	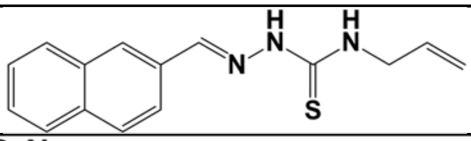	—	nd
138	136	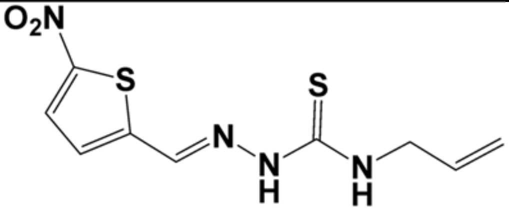	—	
139	135	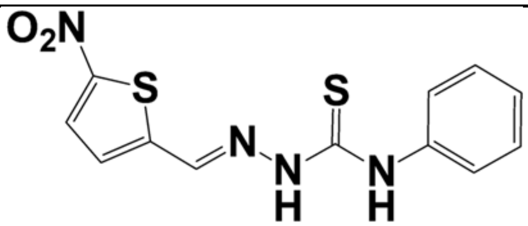	—	
140	280	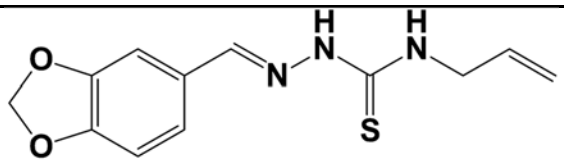	—	
141	900		—	nd

142	1368	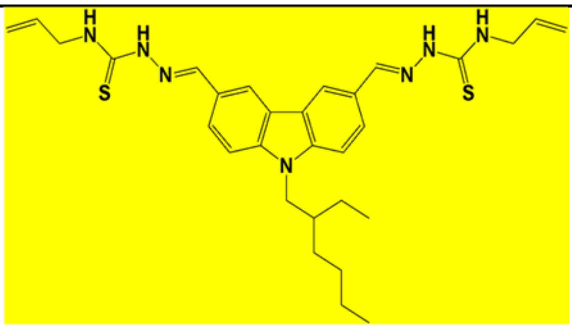	nd	+
143	307		—	nd
144	281		—	nd
145	1260		—	nd
146	1154	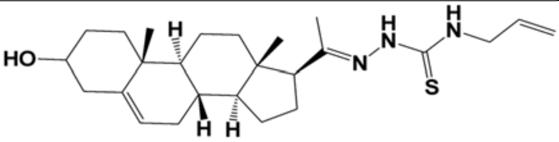	—	nd
147	1383	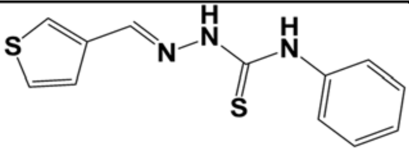	nd	—
**TRIAZINES**				
148	365	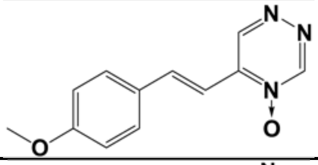	—	nd
149	641	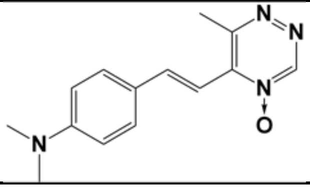	—	nd
**CURCUMINOIDS**				

150	906	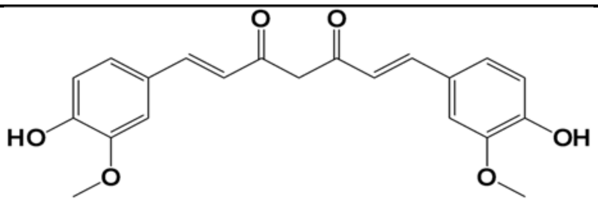	nd	—
151	793		—	nd
152	1246		+	+
153	1247		—	nd
154	1387		—	nd
155	796		—	nd
156	1248		+ / −	nd
157	1018		—	nd
158	1245		—	nd
159	1223		—	nd
160	894		—	nd

161	893		—	nd
162	152		—	nd
163	728		—	nd
164	1271		—	nd
165	731		—	nd
166	151		—	nd
167	885		—	nd
168	881		—	nd
169	150		—	nd

170	692		—	nd
171	690		—	nd
172	1265		—	nd
173	1266		—	nd
174	1267		—	nd
175	1268		+ / −	nd
176	1269		—	nd
177	1254		—	nd

178	1270		—	nd
179	1019		—	nd
180	1256		+	nd
181	1257		+	nd
182	1262		+ / −	+ / −
183	1259		—	nd
184	809		—	nd
**THIAZOLES**				
185	MMV676411			
186	288		—	nd

187	1126	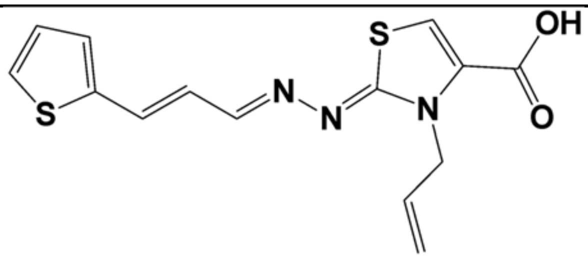	—	
188	910	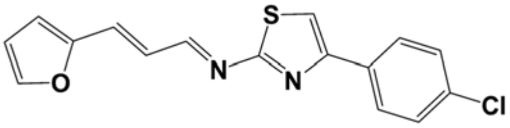	—	
189	259	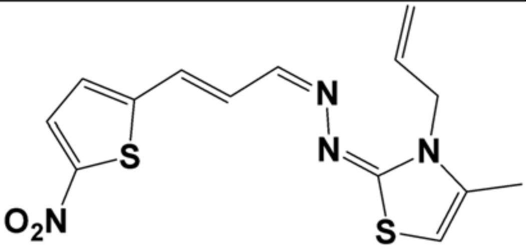	—	
190	1099	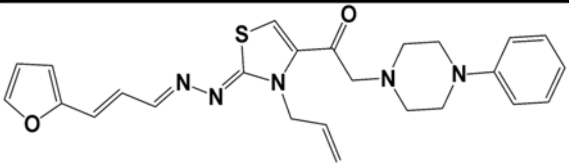	—	
191	1385	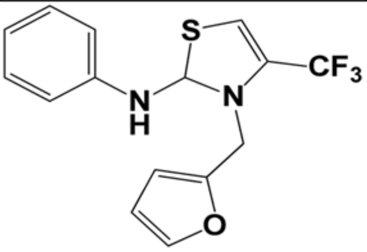	nd	—
192	1310	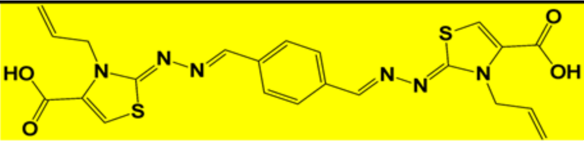	nd	+
193	273		—	nd
194	907		+	nd
195	1090		—	nd
196	266		—	nd

197	871		—	nd
198	276		—	nd
199	264		—	nd
200	314		—	nd
201	1364		—	nd
202	1365		—	nd
203	1376		—	nd
204	1139		—	nd

205	1146		—	nd
206	1153		—	nd
207	1311		—	nd
208	1312		—	nd
209	1118		—	nd
210	789		—	+
211	272		—	nd
212	785		—	+

213	781		—	+
214	1258		—	nd
215	1098		—	nd
216	876		—	nd
217	1128		+	+
218	784		—	+

219	1136		+	+
220	872		—	nd
221	1138		—	+
222	1137		—	nd
223	875		—	nd
224	1133		—	nd
225	1100		—	nd
226	143		—	nd

227	1144		—	nd
228	908		—	nd
229	813		—	nd
230	306		—	+
231	1132		—	nd
232	274		—	nd
233	313		—	Solubility problems
234	904		—	nd

235	1111		—	nd
236	1114		—	nd
237	1115		—	nd
238	1116		—	nd
239	1117		—	+
240	1124		—	nd
241	1131		—	nd

242	810		—	nd
243	791		—	nd
244	270		—	nd
245	1261	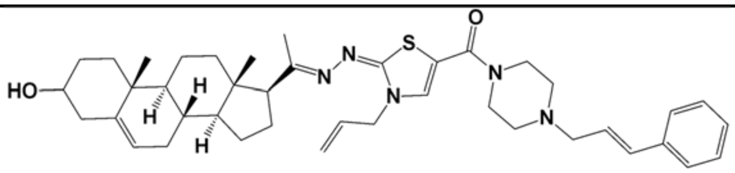	—	nd
246	257		nd	+
247	282		nd	+
248	294		nd	—

249	295		nd	—
250	305		nd	—
251	779		nd	—
252	782		nd	—
253	814		nd	—
254	873		nd	—
255	876		nd	—

256	1093		nd	—
257	1094		nd	—
258	1134	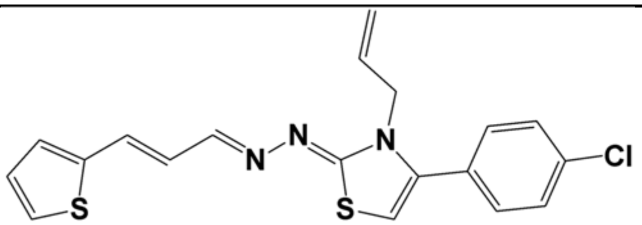	nd	—
259	783	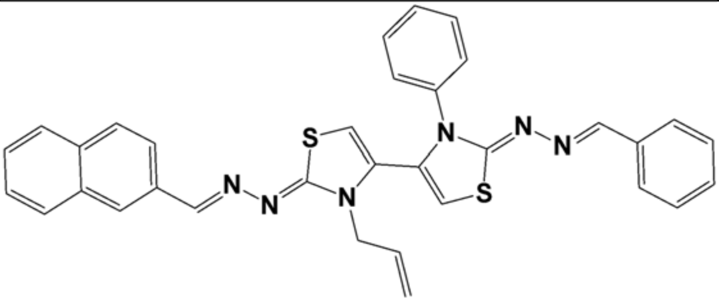	—	nd
260	1112	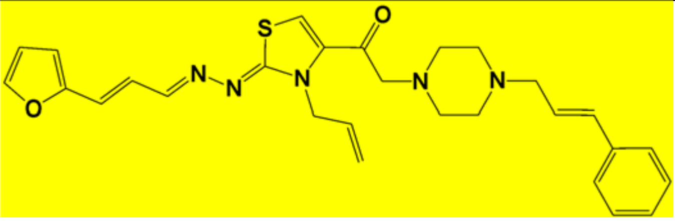	—	+
261	1263	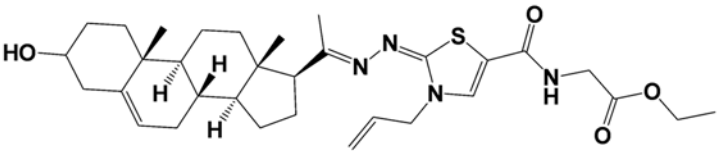	—	nd
262	1103	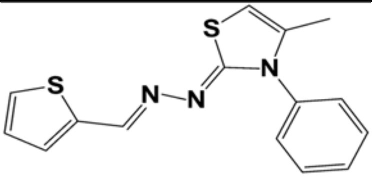	—	nd

263	285	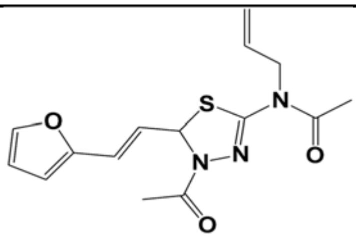	—	nd
264	289		—	+
**POLYAMINES**				
265	MMV026468	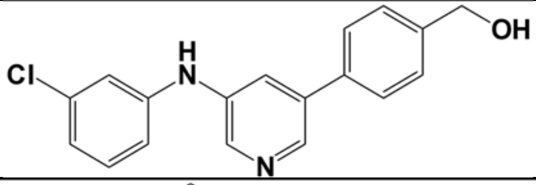	—	nd
266	MMV023953	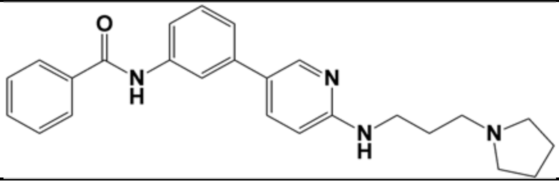	—	nd
267	MMV011511		—	nd
268	MMV024829		—	nd
269	MMV024035			
270	MMV026490			

271	MMV675998			
272	MMV659010			
273	874		—	+
274	1382		—	nd
275	912		—	+
276	903		—	+
277	1102		—	nd
278	MMV024406			

279	MMV023233			
280	MMV085071			
281	MMV659004			
282	MMV676260			
283	MMV688364			
**FLAVONES**				
284	727	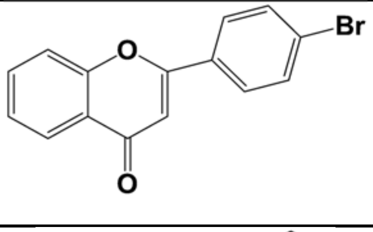	—	nd
285	148	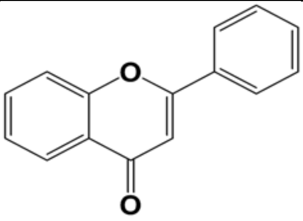	—	nd
286	1264	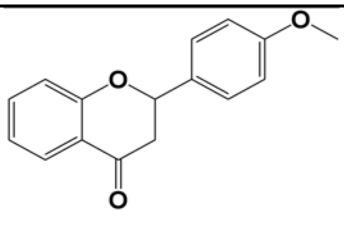	—	nd

287	882	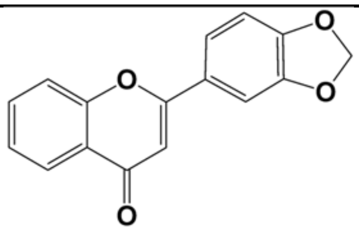	—	
**SELENOCOMPOUNDS**				
288	1378		—	nd
289	1219		—	
290	1097	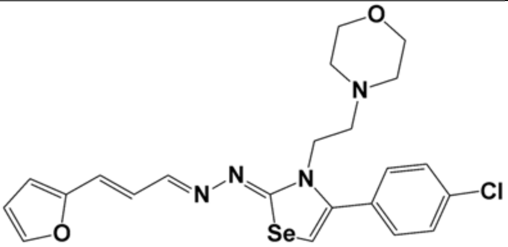	—	
291	1222		—	nd
292	1147		—	nd
**NOT CLUSTERED COMPOUNDS**				
293	1397		—	nd
294	63		—	+

295	256		—	nd
296	1087	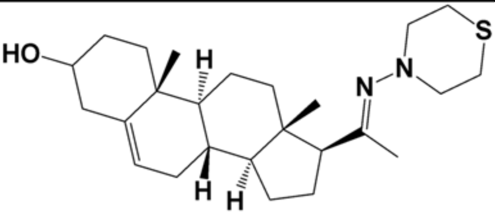	—	
297	879	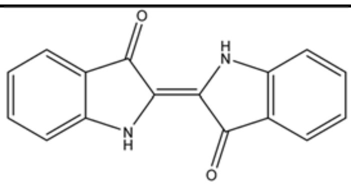	—	nd
298	878	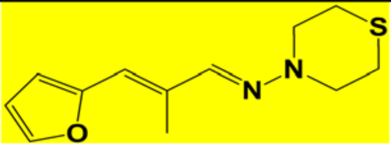	+	+
299	1122	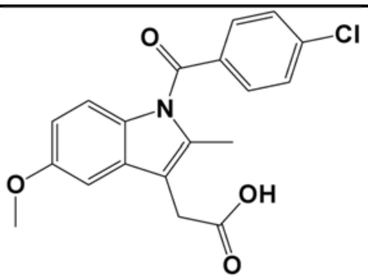	—	
300	1105	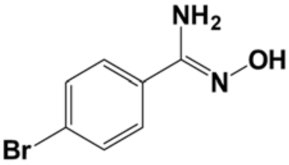	—	
301	1085	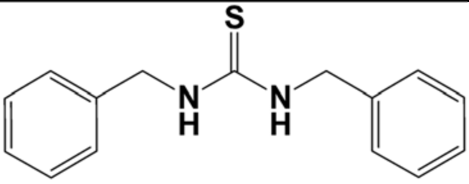	—	
302	1140	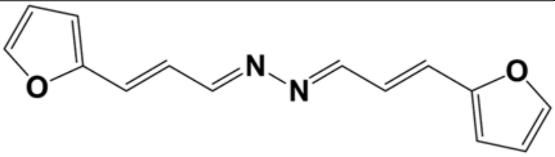	—	
303	1104	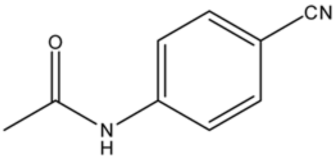		

304	1408			
305	1129	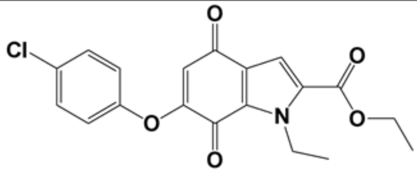	—	
306	237	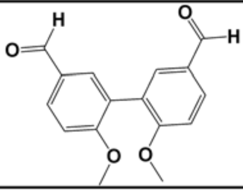	—	
307	242	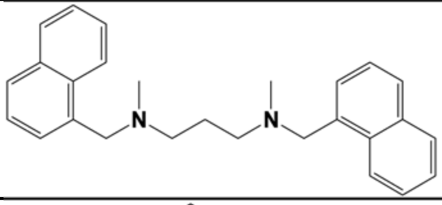	—	
308	1101	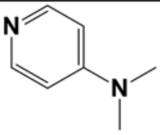	—	
309	379	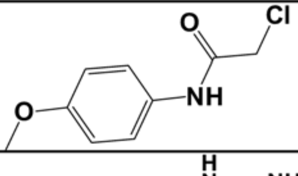	—	
310	675	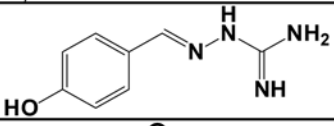	+	—
311	771	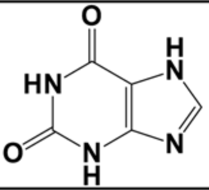	—	
312	716	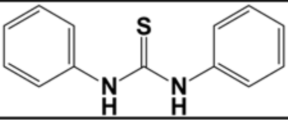	—	
313	718	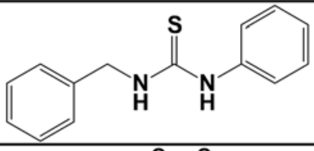	—	
314	249	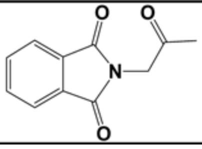	—	

315	659	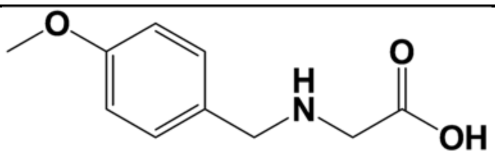	—	
316	MMV00 7471			
317	MMV67 6064			
318	MMV68 8941			
319	MMV67 6162			
320	MMV67 6008			
321	MMV67 6269			

322	MMV020081	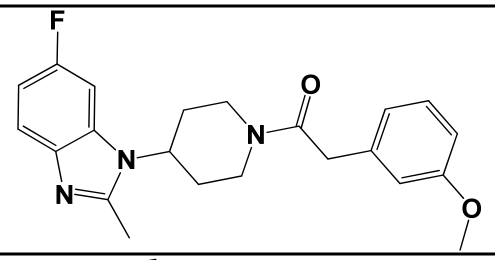		
323	MMV026550	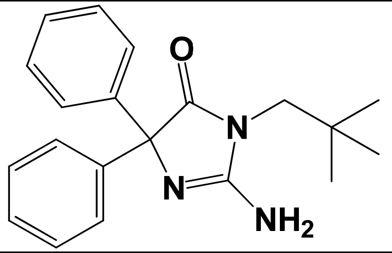		
324	MMV675995	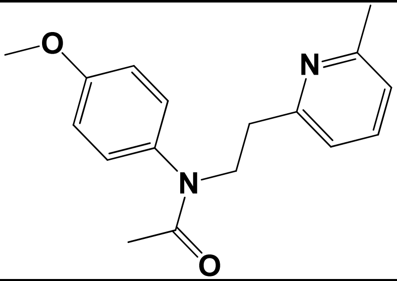		
325	MMV688274	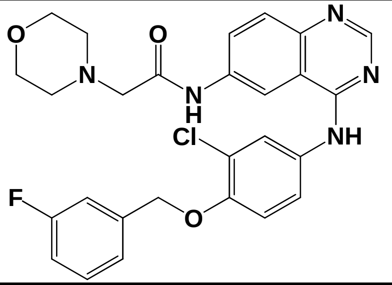		
326	MMV085230	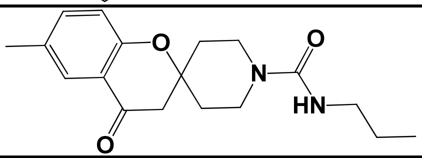		
327	MMV032995	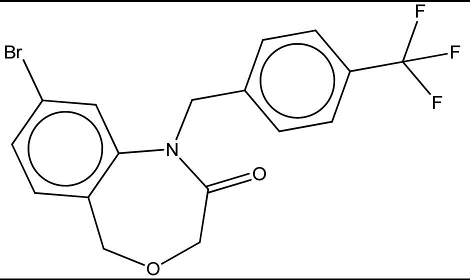		
328	MMV688279	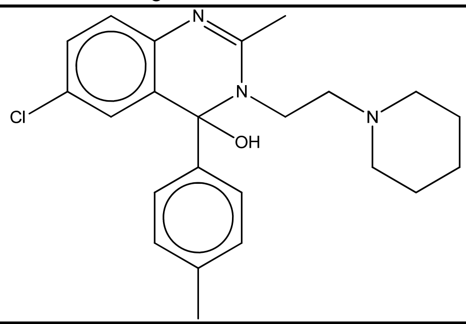		

329	MMV68 8271	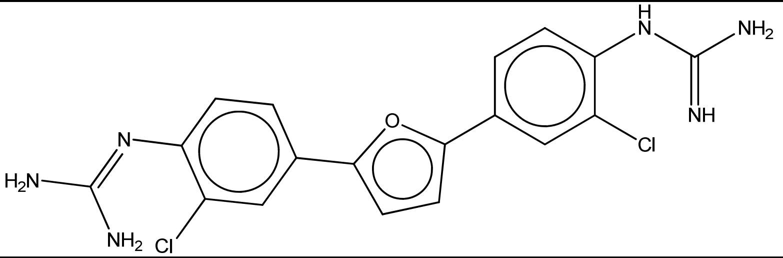		
330	MMV01 9790	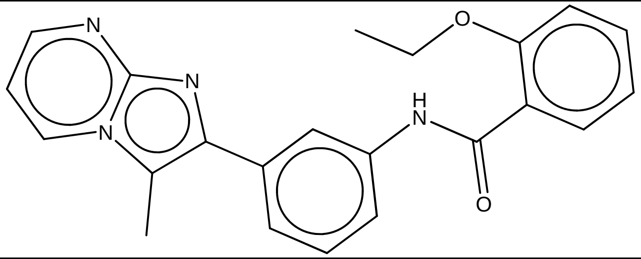		
331	MMV01 1765	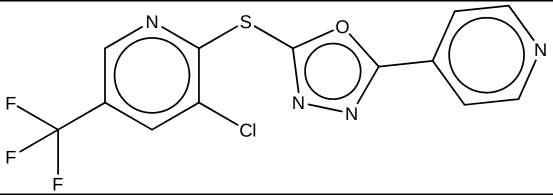		
332	MMV02 4937	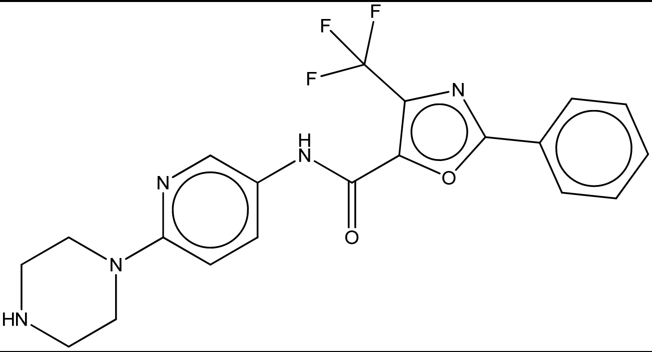		
333	MMV08 5499	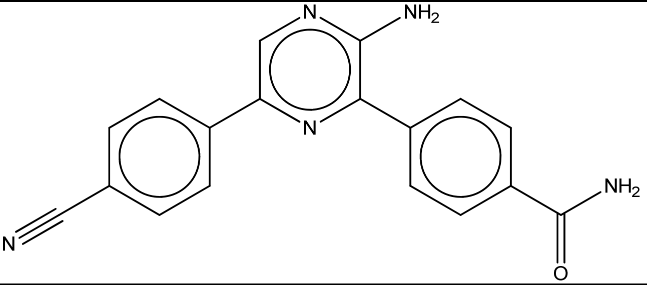		
334	MMV02 3985	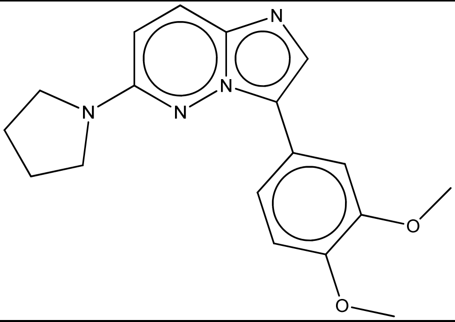		
335	MMV02 4195	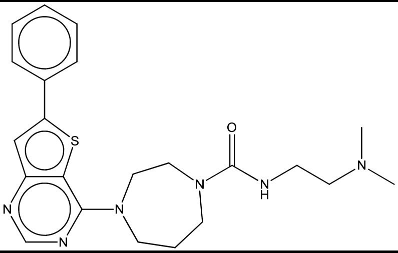		

336	MMV676063	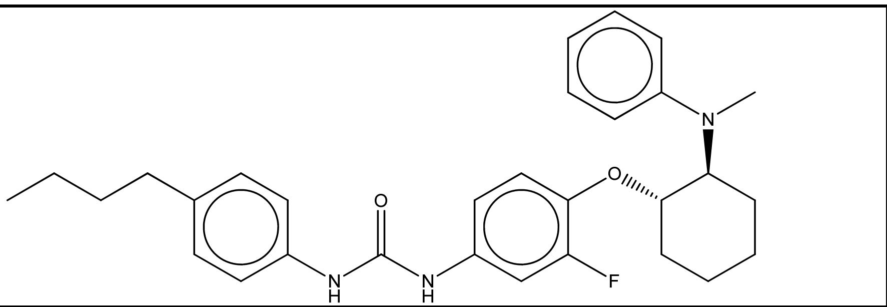		
337	MMV676186	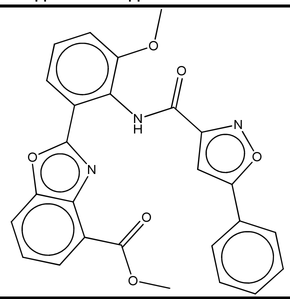		
338	MMV687812	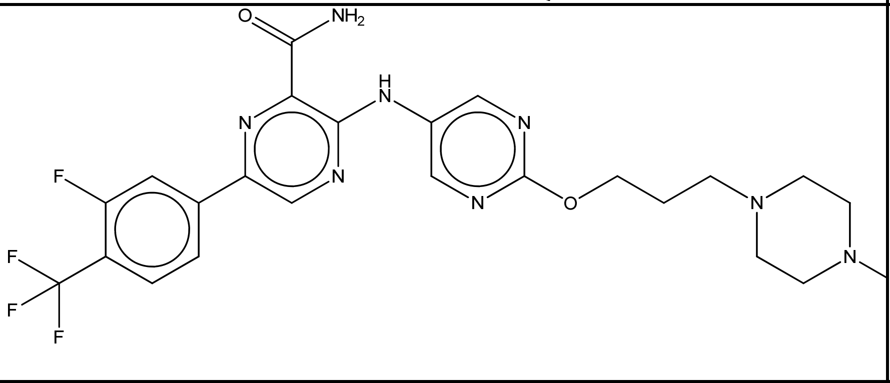		
339	MMV007803	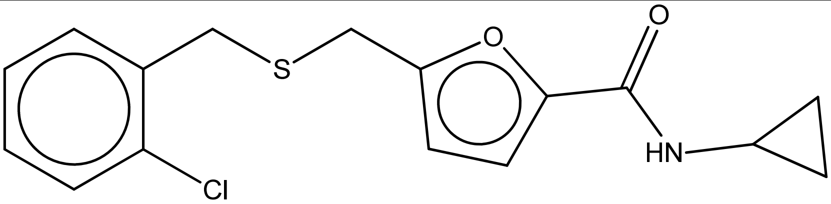		
340	MMV676877	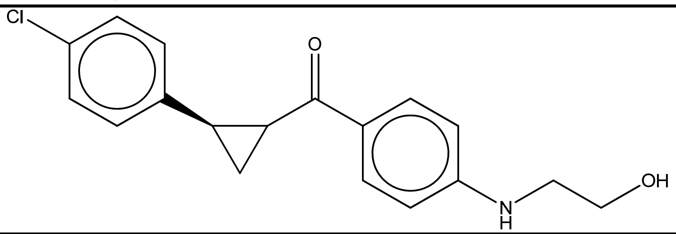		
341	MMV676881	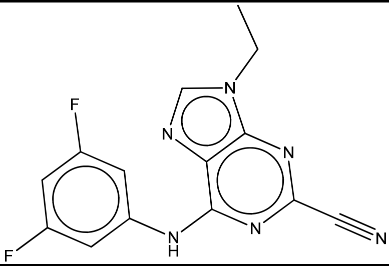		
342	MMV024443	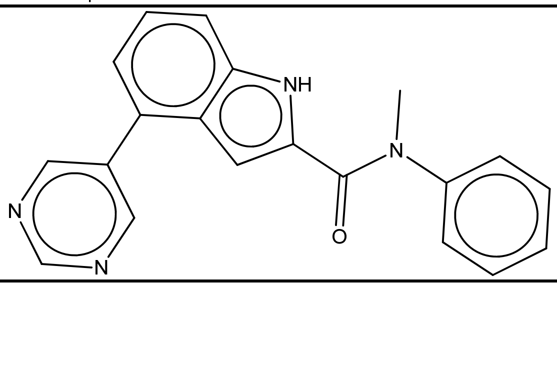		

343	MMV68 8469	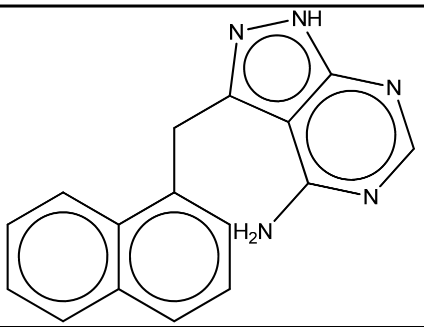		
344	MMV02 3388	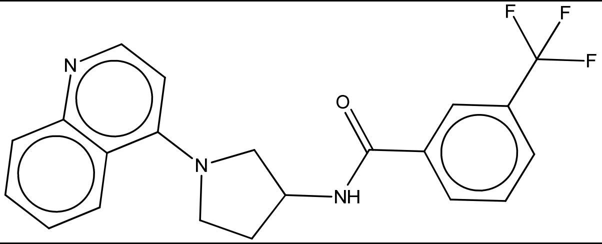		
345	MMV67 5968	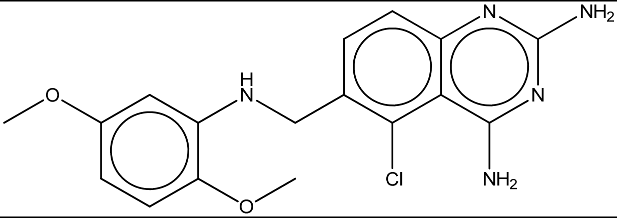		
346	MMV67 5996	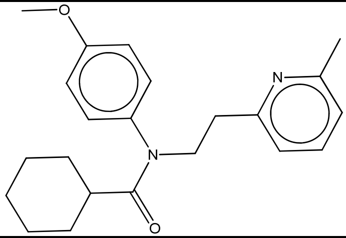		
347	MMV68 8980	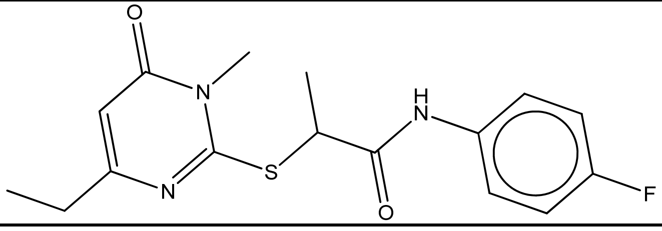		

- + or – means in the case of assemble assay activity or not activity at 50μM. in the binding assay, means binding or not binding respectively. ** is not determined

Table S2. Cytotoxicity assay in mammalian cells

Compound (at 100μM)	Murine macrophages (grown %)	VERO (grown %)
878	98±8	100±8
1136	95±7	100±9
1246	79±9	89±5
1310	98±7	95±7

Figure S1. Output of the search using Chem finder from Chem Office software.

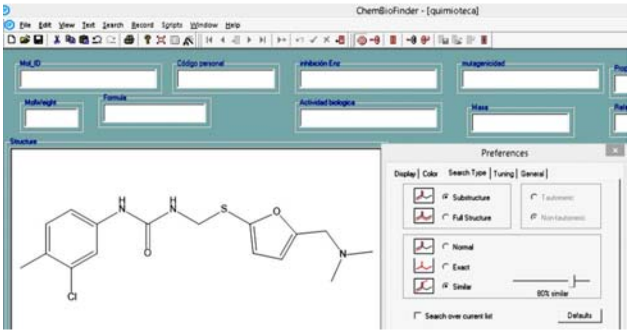

Supplement: Supplementary file 1 [file pharmaceuticals-11-00067-s001.pdf]
